# Respiratory Pathogen Spectrum Among 12,220 Testing Episodes for Clinically Suspected Acute Respiratory Infection in Predominantly Hospitalized Patients at a Tertiary Hospital in Wuhan, China, 2025: A Retrospective Cross-Sectional Study

**DOI:** 10.3390/pathogens15101064

**Published:** 2026-10-08

**Authors:** Yongfen Xiong, Jiao Wang, Jianhua Zhu

**Affiliations:** Department of Clinical Laboratory, Traditional Chinese and Western Medicine Hospital of Wuhan, Tongji Medical College, Huazhong University of Science and Technology, Wuhan 430030, China; xyf5161@whyyy.com (Y.X.); wj5289@whyyy.com (J.W.)

**Keywords:** respiratory pathogen, multiplex PCR, acute respiratory infection, seasonality, co-detection, logistic regression, Wuhan

## Abstract

Acute respiratory infections (ARI) impose a substantial global disease burden, yet the post-pandemic pathogen spectrum in central China remains incompletely characterized. This single-center retrospective cross-sectional study analyzed 12,220 consecutive testing episodes in which a 12-pathogen multiplex PCR panel was ordered for selected patients with clinically suspected ARI at Wuhan No. 1 Hospital in 2025; 99.7% occurred during hospitalization. Overall pathogen detection was 30.3% (3705/12,220). Human rhinovirus was the most frequently detected pathogen (8.84%), followed by influenza A virus (5.29%), respiratory syncytial virus (4.75%), SARS-CoV-2 (4.13%), and human coronavirus (3.77%). Detection rates varied by age (children 60.4% vs. adults 23.3% and elderly 24.6%) and season (autumn 34.1%, winter 33.1%, spring 29.2%, summer 25.4%), with a monthly peak in November (39.3%) and trough in August (21.5%). Co-detection of two or more pathogens occurred in 343 positive episodes (9.26%), with HRV plus RSV being the most common pairing (12.8%). In multivariable analysis, clinical department (pediatrics aOR 5.84, 95% CI 3.69–9.49; hematology aOR 2.19, 95% CI 1.76–2.74) and season (autumn aOR 1.57, 95% CI 1.39–1.77) were independently associated with pathogen detection, whereas sex was not. The strong pediatric age association (aOR 4.99, 95% CI 4.42–5.65) was attenuated to non-significance after adjustment for department (aOR 1.30, 95% CI 0.82–2.00), reflecting near-complete overlap between young age and pediatric attendance, which precludes separation of the two effects; within childhood, detection nevertheless decreased with increasing age (*p* for trend < 0.0001). These findings characterize pathogen detection among predominantly hospitalized patients with clinically suspected ARI at a single tertiary center and may inform local diagnostic stewardship and infection-control practice.

## 1. Introduction

Acute respiratory infections (ARIs), which span a clinical spectrum from self-limiting upper respiratory tract illness to life-threatening lower respiratory tract disease, are among the most common infectious diseases worldwide, causing substantial morbidity, mortality, and healthcare expenditure across all age groups [1]. In China, acute respiratory infections remain one of the leading reasons for seeking medical care, accounting for a substantial share of outpatient and inpatient visits across all age groups [2]. Upper respiratory tract infections (URTIs), which encompass the common cold, pharyngitis, sinusitis, and laryngitis, are by far the most frequently encountered acute illnesses in outpatient and primary care settings and represent the leading cause of acute disease incidence globally. According to the Global Burden of Disease Study 2021, there were an estimated 12.8 billion new episodes of URTIs worldwide in 2021, with an incidence rate of 162,485 per 100,000 population [3]. Lower respiratory infections, although far less frequent than upper respiratory infections, remain a leading infectious cause of death worldwide, with an estimated 344 million episodes and 2.18 million deaths globally in 2021 [4]. Although the majority of ARIs are mild and self-limiting, they can occasionally progress to lower respiratory tract infections, including pneumonia, acute respiratory distress syndrome, and respiratory failure, particularly in young children, older adults, and immunocompromised individuals [5,6].

The etiology of ARIs is diverse, encompassing viruses, atypical bacteria, and fungi. Common viral pathogens include influenza A and B viruses (IAV and IBV), respiratory syncytial virus (RSV), human rhinovirus (HRV), human coronavirus (HCoV), human parainfluenza virus (HPIV), human adenovirus (HAdV), human metapneumovirus (hMPV), and human bocavirus (HBoV), while atypical bacterial pathogens such as *Mycoplasma pneumoniae* (MP) and *Chlamydia pneumoniae* (CP) are also recognized contributors [6,7,8]. The prevalence and seasonal patterns of these pathogens vary considerably by geography, climate, demography, and population immunity [6,9]. The COVID-19 pandemic and the associated non-pharmaceutical interventions profoundly altered the epidemiology of respiratory pathogens, suppressing influenza and RSV circulation during 2020–2022, followed by heterogeneous resurgence patterns after the relaxation of control measures [10]. Because many respiratory pathogens cause clinically indistinguishable syndromes and inappropriate antibiotic use remains common in patients with acute respiratory illness, rapid and accurate etiological diagnosis has become increasingly important [11]. Syndromic multiplex PCR panels have become indispensable tools for rapid and simultaneous detection of multiple respiratory pathogens, improving diagnostic precision and enabling robust pathogen-spectrum surveillance [12,13].

Wuhan, the largest city in central China and a major transportation hub, plays a pivotal role in the spread of respiratory pathogens due to its high population mobility and extensive regional connections. Despite heightened research interest following the COVID-19 pandemic, comprehensive data on the post-pandemic respiratory pathogen spectrum in Wuhan remain limited, particularly for the year 2025. Existing local studies have focused predominantly on hospitalized children, severe acute respiratory infections, or single pathogens such as SARS-CoV-2 or IAV [14,15]. Comprehensive all-age molecular data on clinically suspected acute respiratory infections in this setting, incorporating simultaneous detection of multiple common respiratory pathogens, are still lacking. Furthermore, the patterns of co-detection and the factors independently associated with pathogen detection have not been systematically evaluated in this setting. Moreover, because molecular testing in routine care is ordered at the discretion of treating physicians rather than by a standardized protocol, the clinical setting in which testing is ordered may itself shape observed detection rates—an issue that has received little attention in existing pathogen-spectrum studies.

To address these gaps, we conducted a large retrospective cross-sectional study using data from testing episodes in which multiplex PCR testing for 12 respiratory pathogens was ordered for selected patients with clinically suspected acute respiratory infection (ARI) at Wuhan No. 1 Hospital during 2025. The aims were to characterize the overall pathogen spectrum, to analyze seasonal and age-stratified distribution patterns, to describe co-detection patterns, and to identify factors independently associated with respiratory pathogen detection using multivariable logistic regression. We hypothesized that pathogen detection rates would vary significantly by season, age, and clinical department, and that multivariable adjustment would reveal department-specific and seasonal risks independent of demographic confounders.

## 2. Materials and Methods

### 2.1. Study Design and Setting

This was a single-center retrospective cross-sectional study conducted at Wuhan No. 1 Hospital, a tertiary-grade general hospital in Wuhan, Hubei Province, China. The study period covered 1 January 2025 to 31 December 2025, spanning all four seasons. Wuhan (30°35′ N, 114°17′ E) is the capital of Hubei Province and the most populous city in central China, with a resident population exceeding 13 million. Wuhan No. 1 Hospital is designated as a national sentinel hospital for influenza and SARS-CoV-2 surveillance and for multiple respiratory pathogen surveillance. The present analysis, however, is based on all routine clinical multiplex PCR tests rather than the sentinel surveillance sampling protocol, and should therefore not be interpreted as sentinel surveillance data.

### 2.2. Study Population and Sample Selection

**Figure 1:**
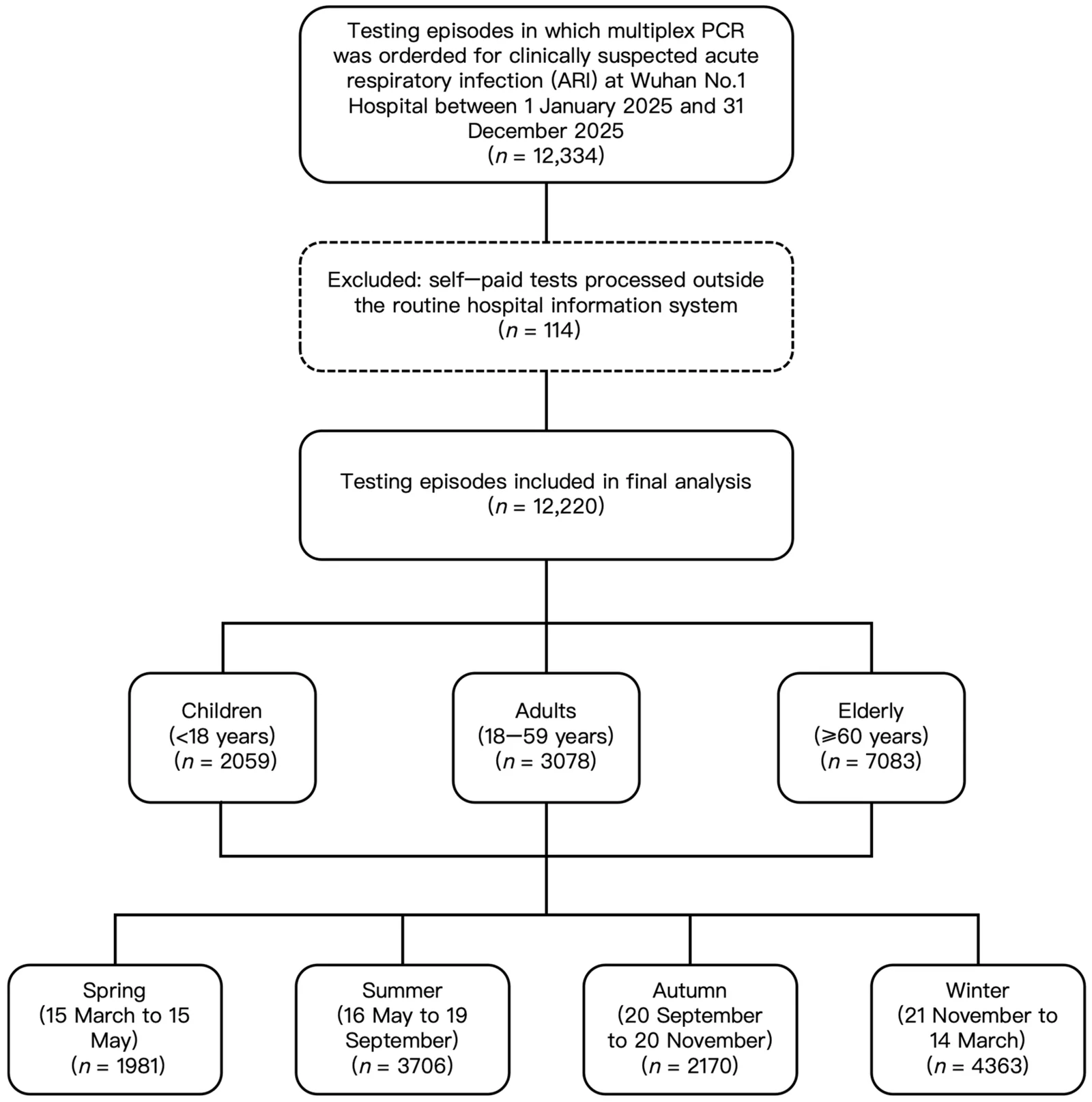
Flowchart of patient selection and study enrollment. A total of 12,334 testing episodes in which multiplex PCR was ordered for clinically suspected acute respiratory infection (ARI) at Wuhan No. 1 Hospital between 1 January and 31 December 2025 were initially screened. After excluding 114 self-paid tests processed outside the routine hospital information system, to which no medical record number, patient type, department, or diagnosis could be linked, 12,220 testing episodes were included in the final analysis. Episodes were stratified by age group and by season. Seasons were defined as described in Section 2.4.

This analysis included all consecutive respiratory multiplex PCR tests performed at Wuhan No. 1 Hospital between 1 January and 31 December 2025. The panel was ordered at the discretion of the treating physician for clinically suspected acute respiratory infection (ARI) in the course of routine clinical care. The analysis cohort is therefore defined by testing rather than by an independently verified clinical syndrome, and all proportions reported are detection proportions among testing episodes. The unit of analysis was the testing episode rather than the unique patient, because each episode corresponds to a distinct specimen and a distinct decision to test, and because the quantity of interest is the yield of testing—the proportion of specimens in which a panel target is detected—which is conventionally reported per specimen and is the relevant denominator for laboratory workload, diagnostic stewardship and surveillance comparisons. The 12,220 episodes were contributed by 10,028 unique patients, of whom 1300 (13.0%) contributed two or more episodes (2192 additional episodes). A further 114 tests were excluded because they were self-paid tests processed outside the routine hospital information system; no medical record number, patient type, clinical department, or diagnosis could be linked to these tests, precluding their assignment to any analysis stratum (Figure 1).

### 2.3. Pathogen Detection

Respiratory specimens (oropharyngeal or nasal swabs) were collected as part of routine clinical care. Immediately after collection, each swab was placed into a sample preservation tube meeting the specifications of the assay manufacturer and delivered to the clinical laboratory without delay. Specimens that could not be tested immediately were stored at 4 °C, and all specimens were processed within 6 h of collection. Nucleic acid was extracted using the BioCreate nucleic acid extraction system (Qtract-32, Guangzhou BioCreate Biotechnology Co., Ltd., Guangzhou, China) with the corresponding extraction reagents, and amplified on a SLAN-96 real-time fluorescence quantitative PCR system (Hongshi Medical Technology, Shanghai, China) using a commercial 12-pathogen respiratory nucleic acid detection kit (Guangzhou BioCreate Biotechnology Co., Ltd., Guangzhou, China) targeting HRV, IAV, IBV, RSV, SARS-CoV-2, HCoV, HPIV, HAdV, hMPV, HBoV, MP, and CP. Each reaction contained an endogenous internal amplification control, and negative and positive controls were run in every batch. A run was considered valid when the internal control amplified with a Ct ≤ 42, the negative control showed no amplification within 40 cycles, and the positive control amplified with a Ct ≤ 40; otherwise, the specimen was re-tested. According to the manufacturer’s instructions, a target was interpreted as positive when the Ct value was ≤ 40.

### 2.4. Variable Definitions

The outcome variable was molecular pathogen detection (positive if any of the 12 targets was detected within a testing episode). Because a patient may contribute more than one episode and an episode may yield more than one pathogen, results are reported at three levels: testing episodes with at least one pathogen detected, individual pathogen detections, and co-detections. An episode with one pathogen detected was classified as single-pathogen detection, with two as dual-pathogen detection, and with three or more as multiple-pathogen detection. A patient’s first testing episode was defined as the earliest testing episode recorded under that patient’s registration number. Age was categorized as children (<18 years), adults (18–59 years), and elderly (≥60 years). Seasons were defined following the local climatic seasons of Wuhan: spring (15 March to 15 May), summer (16 May to 19 September), autumn (20 September to 20 November), and winter (21 November to 14 March). These fixed calendar boundaries approximate Wuhan’s long-term climatic season onsets under Chinese national standard GB/T 42074-2022 [16] (spring/autumn, 10–22 °C; summer, ≥22 °C; winter, <10 °C; 5-day running means), ensuring reproducibility; monthly analyses were performed to assess the robustness of the seasonal patterns. The clinical department was classified as Pediatrics, Hematology, Nephrology, Cardiology, Respiratory, Rheumatology, or Others; the neonatology unit and pediatric emergency service (*n* = 6) were grouped under Pediatrics.

### 2.5. Statistical Analysis

Categorical variables are expressed as counts and percentages. All variables included in the analyses were completely recorded; analyses were therefore performed on complete cases, and no imputation was required. Two-sided 95% confidence intervals for single proportions were calculated with the Wilson score method throughout. Because 1300 patients (13.0%) contributed more than one episode, 2192 of the 12,220 episodes (17.9%) were not independent observations; a sensitivity analysis restricted to each patient’s first testing episode (*n* = 10,028) was performed to examine whether repeated testing by the same patient influenced the findings. Monthly detection rates were calculated for all episodes and, in the sensitivity analysis, for the subset of first episodes only.

Group comparisons of positivity rates were performed using the chi-square (χ^2^) test for contingency tables; Fisher’s exact test was substituted for the chi-square test whenever the minimum expected cell count fell below 5. Pairwise comparisons among the three age groups were adjusted using the Bonferroni method. Exploratory pathogen-specific comparisons across seasons and age groups were corrected for multiple testing using the Benjamini–Hochberg false discovery rate (FDR) procedure. Each correction family comprised the 12 pathogen-specific tests within a given comparison (12 across seasons and 12 across age groups); the global comparisons of overall positivity across seasons and across age groups were not included in these families and are reported uncorrected. Detection across the five ordered childhood age bands was summarized with a Cochran–Armitage chi-square test for trend.

Multivariable logistic regression was used to estimate adjusted odds ratios (aORs) and 95% confidence intervals (CIs) for factors associated with respiratory pathogen detection; the 95% CIs reported for odds ratios were Wald intervals derived from the standard errors of the fitted logistic regression models. Crude odds ratios were obtained from single-predictor logistic regression models in which each variable was entered separately. The reference categories were adults (age group), female (sex), summer (season), and rheumatology (clinical department). Two logistic regression models were fitted: Model 1 included age group, sex, and season, and Model 2 additionally included clinical department. Model 2, which included all candidate predictors, was treated as the primary model; no adjustment for multiplicity was applied to the regression coefficients, which are reported with nominal 95% CIs. Model discrimination was assessed by the area under the receiver operating characteristic curve (AUC) for both models, and goodness of fit was evaluated for Model 2 using the Hosmer–Lemeshow test, with episodes grouped into deciles of predicted probability. Because age group and clinical department were a priori expected to be closely related, multicollinearity among model covariates was assessed by calculating generalized variance inflation factors (GVIFs) and their dimensionality-adjusted values (GVIF^1/(2 × *Df*)^), with values below 5.0 considered acceptable. In addition, the cross-distribution of age group and clinical department was tabulated, and a post hoc sensitivity analysis was performed among children attending non-pediatric departments to examine the extent to which the age effect could be separated from the testing setting; in that comparison, detection in non-pediatric settings was standardized for age by direct standardization, using the age-specific rates observed among pediatric episodes as the reference; the standardized detection ratio and its 95% CI were derived from the Poisson distribution of the observed number of positive episodes.

Statistical analyses were performed using GraphPad Prism version 9.5.0 (GraphPad Software, San Diego, CA, USA) and R version 4.6.1 (R Foundation for Statistical Computing, Vienna, Austria). A two-sided *p* value < 0.05 was considered statistically significant. Significance levels are denoted as * *p* < 0.05, ** *p* < 0.01, *** *p* < 0.001, and **** *p* < 0.0001.

### 2.6. Ethics Statement

The study was conducted in accordance with the Declaration of Helsinki and was approved by the Ethics Committee of Wuhan No. 1 Hospital on 18 August 2026 (Kelun [2026] No. 125). Because this was a retrospective analysis using de-identified data, the requirement for informed consent was waived by the Ethics Committee. The study protocol was developed after the end of the study period, and data extraction and analysis commenced only after the above approval had been obtained; no data were accessed for research purposes before approval.

## 3. Results

### 3.1. Study Population Characteristics

A total of 12,220 testing episodes were included in the final analysis after excluding 114 self-paid tests processed outside the routine hospital information system. These episodes were contributed by 10,028 unique patients identified by registration number, of whom 1300 (13.0%) contributed more than one episode. Of the included episodes, 12,189 (99.7%) were ordered during hospitalization, 28 (0.23%) in outpatient clinics, and 3 (0.02%) in the emergency department (Supplementary Table S1). Specimens were oropharyngeal swabs in 12,196 episodes (99.8%) and nasal swabs in 24 (0.2%) (Supplementary Table S1), the latter obtained mainly in the cardiac intensive care unit and hematology wards from adult or elderly patients. Among the episodes, 6589 (53.9%) were contributed by male patients and 5631 (46.1%) by female patients. By age group, 2059 episodes (16.8%) were from children, 3078 (25.2%) were from adults, and 7083 (58.0%) were from elderly patients. Seasonal distribution was as follows: spring, 1981 (16.2%); summer, 3706 (30.3%); autumn, 2170 (17.8%); and winter, 4363 (35.7%). Overall, 3705 episodes (30.3%) were positive for at least one respiratory pathogen. Among these positive episodes, 3362 (90.7%) had single-pathogen detection, 317 (8.56%) dual-pathogen detection, and 26 (0.70%) had multiple-pathogen detection (Table 1). The 114 excluded self-paid tests, compared with the included cohort, had a higher proportion of children (49.1% vs. 16.8%) and a higher detection rate (53.5% vs. 30.3%). The recorded diagnoses of the included episodes, grouped into four mutually exclusive diagnostic categories, are summarized in Supplementary Table S2. Comorbidity burden increased sharply with age: at least one comorbidity category was recorded in 2.4% (49/2059) of children’s episodes, 52.3% (1610/3078) of adults’ episodes, and 75.2% (5328/7083) of elderly patients’ episodes (57.2% of all episodes; Supplementary Table S3). The distribution of testing episodes across the seven clinical department categories, together with department-specific detection rates, is provided in Table A1.

### 3.2. Overall Detection Rates of Respiratory Pathogens

**Figure 2:**
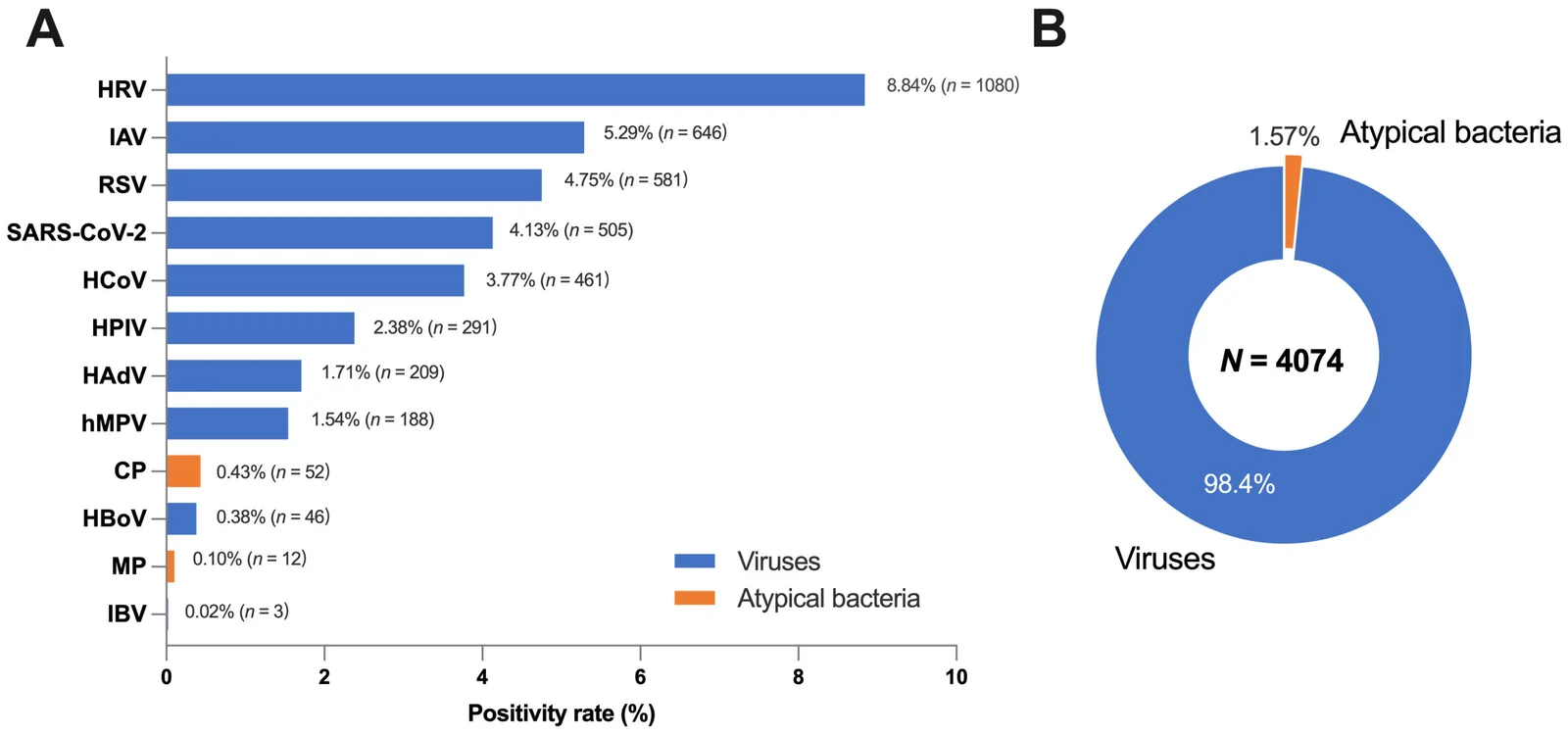
Overall detection rates of 12 respiratory pathogens among testing episodes at Wuhan No. 1 Hospital, 2025 (*N* = 12,220). (**A**) Bar chart showing positivity rates of individual pathogens in descending order. Blue bars represent viruses; orange bars represent atypical bacteria. (**B**) Pie chart showing the proportion of viral vs. atypical bacterial (*Chlamydia pneumoniae* and *Mycoplasma pneumoniae*) detections among all pathogen detections.

Among the 12 pathogens evaluated, HRV was the most frequently detected (8.84%, *n* = 1080), followed by IAV (5.29%, *n* = 646), RSV (4.75%, *n* = 581), SARS-CoV-2 (4.13%, *n* = 505), HCoV (3.77%, *n* = 461), HPIV (2.38%, *n* = 291), HAdV (1.71%, *n* = 209), hMPV (1.54%, *n* = 188), CP (0.43%, *n* = 52), HBoV (0.38%, *n* = 46), MP (0.10%, *n* = 12), and IBV (0.02%, *n* = 3). Among the 4074 individual pathogen detections, 4010 (98.4%) were viral and 64 (1.6%) were atypical bacterial; this composition may reflect the design of the panel, which includes ten viral and two atypical bacterial targets and no typical bacterial pathogens, and does not represent the etiological composition of respiratory infections in the population. The sum of individual pathogen detections (*n* = 4074) exceeded the number of positive episodes (*n* = 3705) because of co-detections (Table 2; Figure 2).

When the analysis was restricted to each patient’s first testing episode (*n* = 10,028; 2192 repeat episodes from 1300 patients excluded), the detection rate was 30.4% (3048/10,028; 95% CI 29.5–31.3), compared with 30.3% (3705/12,220; 95% CI 29.5–31.1) at the episode level, a difference of 0.08 percentage points. The two analyses agreed closely in every stratum examined: differences did not exceed 0.64 percentage points across seasons, 1.70 percentage points across age groups, 2.13 percentage points across months, or 0.60 percentage points for individual pathogens. All 12 pathogens retained their original rank order, and both the November maximum (40.0%) and the August minimum (21.9%) were preserved. Repeated testing by a minority of patients therefore did not materially influence the principal findings (Supplementary Table S4).

### 3.3. Seasonal Distribution

**Figure 3:**
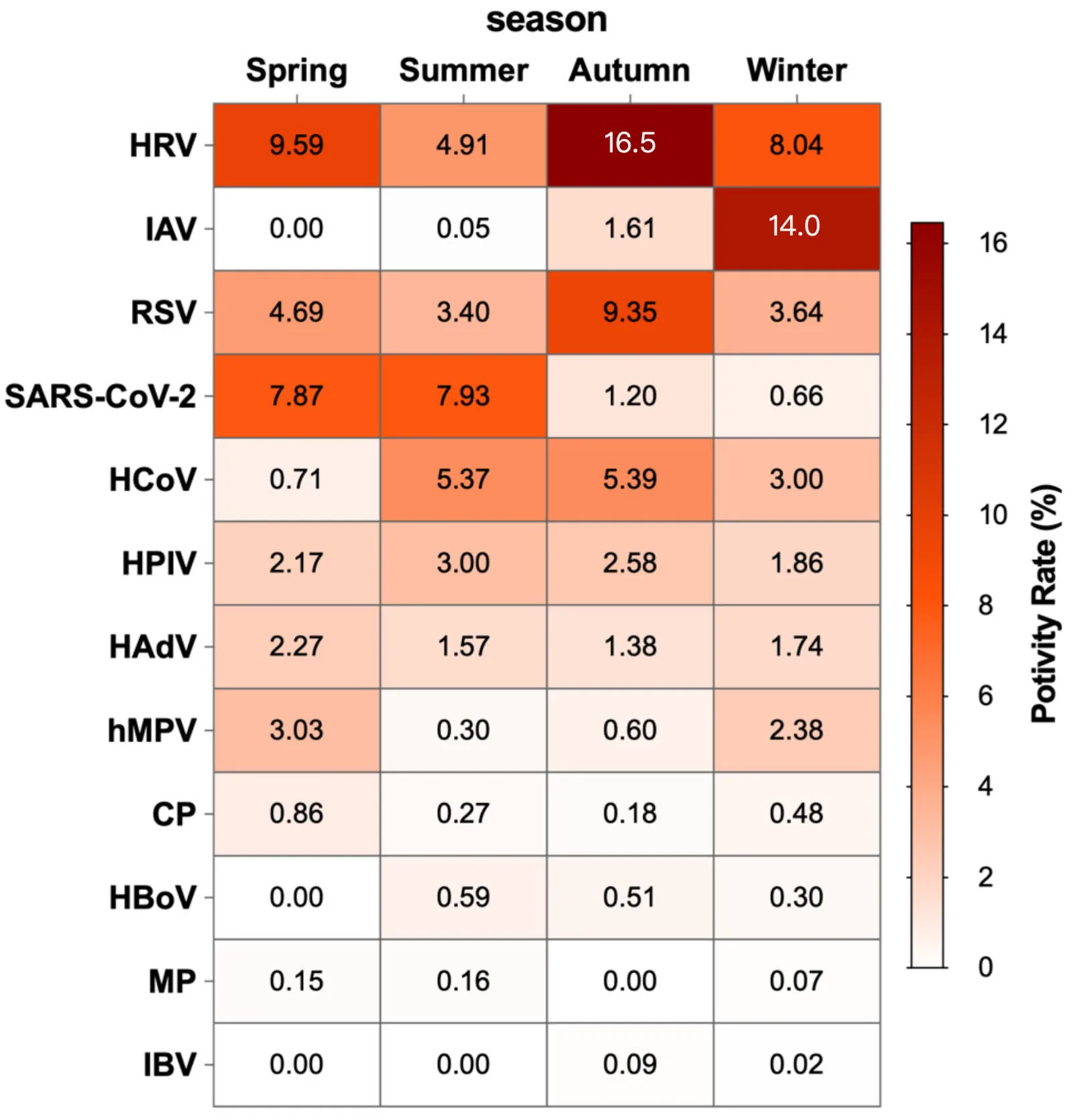
Heatmap of seasonal positivity rates for 12 respiratory pathogens among testing episodes at Wuhan No. 1 Hospital, 2025. Color intensity represents the positivity rate (%) for each pathogen within each season. Pathogens are listed in descending order of overall detection rate. Seasons were defined as described in Section 2.4.

**Figure 4:**
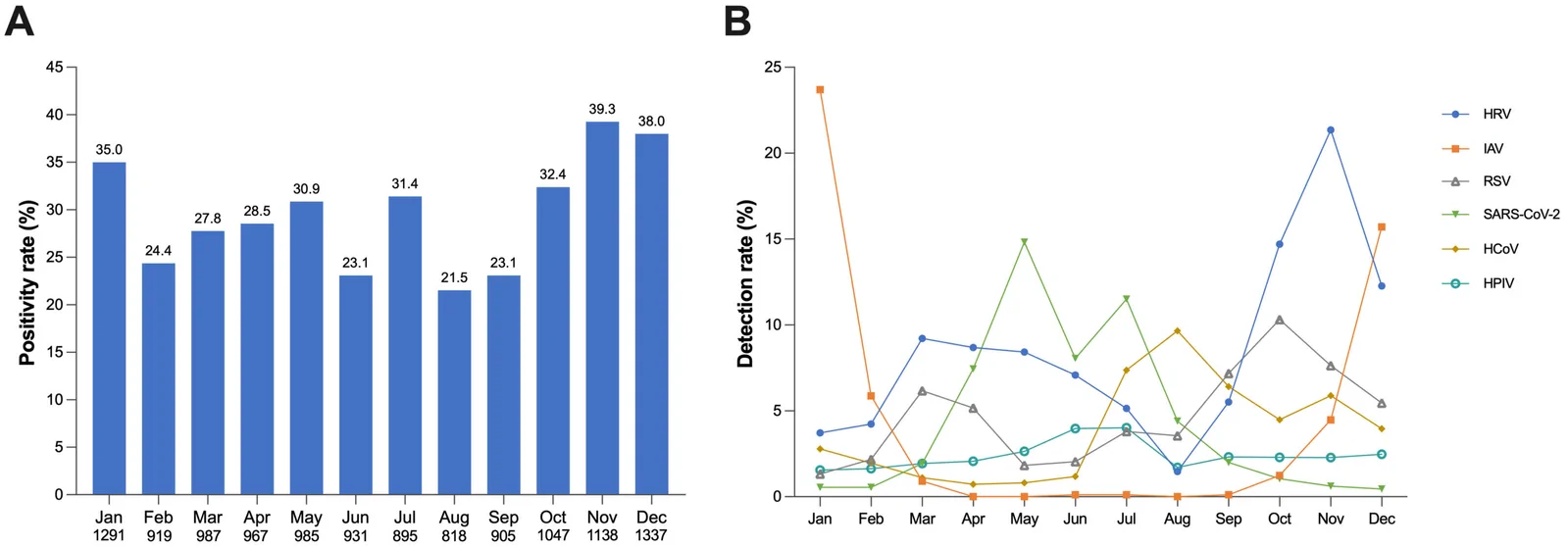
Monthly pathogen detection rates among testing episodes at Wuhan No. 1 Hospital, 2025. (**A**) Monthly overall positivity rate; month labels show the number of testing episodes per month. (**B**) Monthly detection rates of the six most frequently detected pathogens.

The overall pathogen positivity rate differed significantly across seasons (*χ*^2^ = 74.7, *df* = 3, *p* < 0.0001), with the highest rate observed in autumn (34.1%, 741/2170), followed by winter (33.1%, 1444/4363), spring (29.2%, 579/1981), and summer (25.4%, 941/3706). The dominant pathogen varied by season: HRV predominated in spring (9.59%) and autumn (16.5%), SARS-CoV-2 in summer (7.93%), and IAV in winter (14.0%). Notably, the autumn HRV positivity rate (16.5%) was the highest single pathogen-season value observed in the entire dataset, whereas winter IAV (14.0%) reflected a pronounced seasonal influenza peak. SARS-CoV-2 showed relatively stable circulation in spring (7.87%) and summer (7.93%) but declined markedly in autumn (1.20%) and winter (0.66%) (Table 3; Figure 3; Table A2). In exploratory pathogen-specific analyses corrected for multiple testing using the Benjamini–Hochberg false discovery rate procedure, nine of the 12 pathogens showed significant seasonal variation (all except HAdV, IBV, and MP; Supplementary Table S5). Monthly rates confirmed the seasonal pattern, with the highest positivity in November (39.3%, 447/1138) and December (38.0%, 508/1337) and the annual trough in August (21.5%, 176/818) (Figure 4).

### 3.4. Age-Stratified Distribution

**Figure 5:**
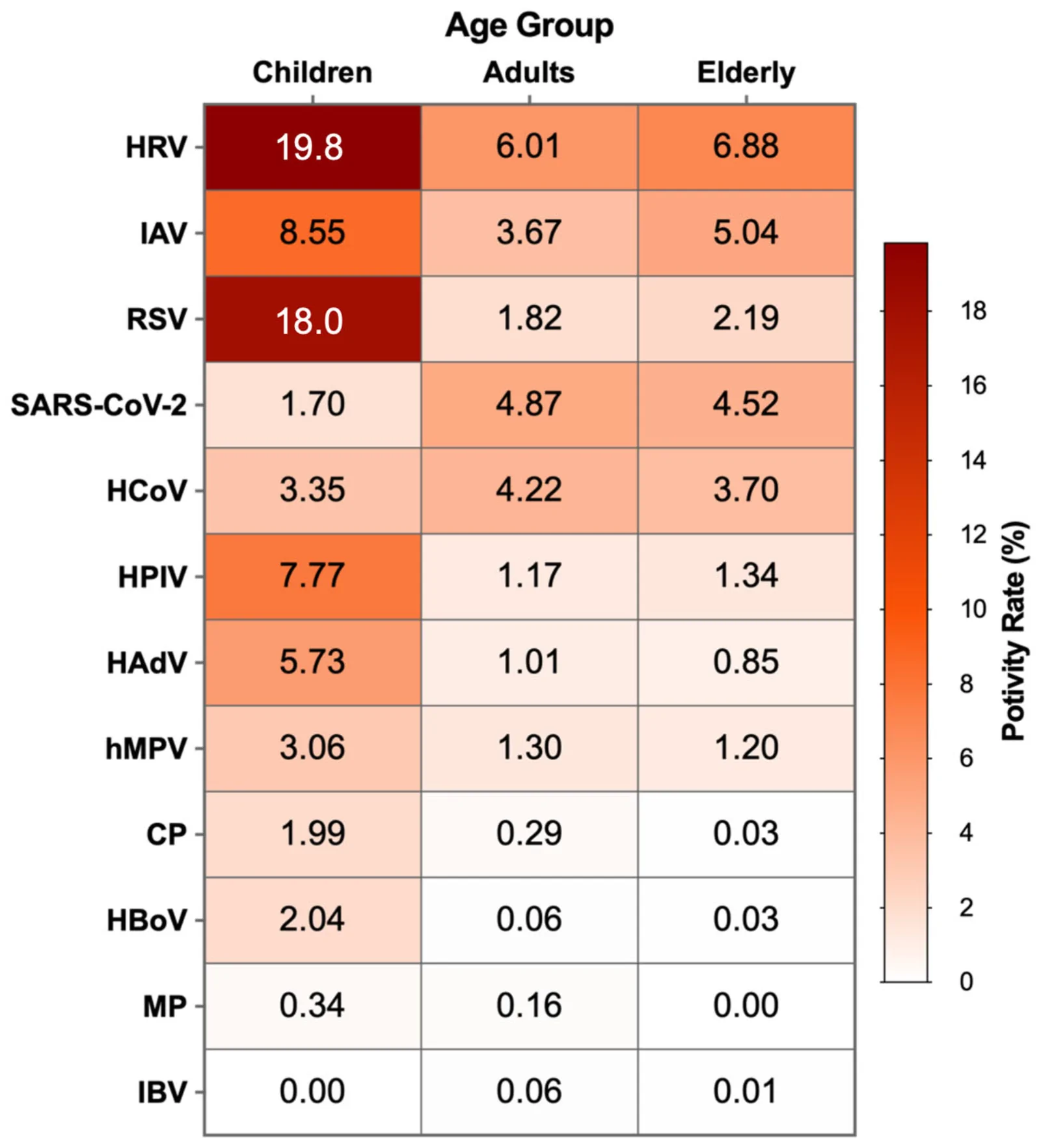
Heatmap of age-stratified positivity rates for 12 respiratory pathogens among testing episodes at Wuhan No. 1 Hospital, 2025. Color intensity represents the positivity rate (%) for each pathogen within each age group. Pathogens are listed in descending order of overall detection rate, consistent with Figure 3. Age groups: Children (<18 years), Adults (18–59 years), Elderly (≥60 years).

Pathogen positivity varied substantially by age group (*χ*^2^ = 1060, *df* = 2, *p* < 0.0001). Children had the highest positivity rate (60.4%, 1244/2059), significantly exceeding both adults (23.3%, 717/3078) and elderly patients (24.6%, 1744/7083) (both *p* < 0.0001 after Bonferroni correction), whereas the difference between adults and elderly patients was not significant (*p* = 0.15). HRV was the dominant pathogen in all three age groups, with positivity rates of 19.8% in children, 6.01% in adults, and 6.88% in elderly patients. RSV showed a marked age gradient, with positivity of 18.0% in children compared with 1.82% in adults and 2.19% in elderly patients. By contrast, SARS-CoV-2 positivity was higher in adults (4.87%) and elderly patients (4.52%) than in children (1.70%). Atypical bacteria (CP and MP) and HBoV were detected almost exclusively in children (Table 4; Figure 5). In exploratory FDR-corrected analyses, ten of the 12 pathogens showed significant age-related variation (all except IBV and HCoV; Supplementary Table S6). However, the small numbers of MP (*n* = 12), HBoV (*n* = 46) and IBV (*n* = 3) detections preclude firm epidemiological conclusions for these pathogens.

Among children, detection declined with increasing age: 66.7% (<1 year), 70.2% (1–2 years), 66.4% (3–5 years), 49.7% (6–11 years), and 32.9% (12–17 years) (Supplementary Table S7). The gradient persisted when the analysis was restricted to the 1955 episodes ordered in pediatric departments, where the testing setting was constant (67.0%, 70.0%, 66.4%, 49.8% and 45.6% for <1, 1–2, 3–5, 6–11 and 12–17 years, respectively; *χ^2^* for trend = 45.7, *df* = 1, *p* < 0.0001; Supplementary Table S7, Panel B), indicating that it is not attributable to differences in testing setting.

### 3.5. Co-Detection Patterns

**Figure 6:**
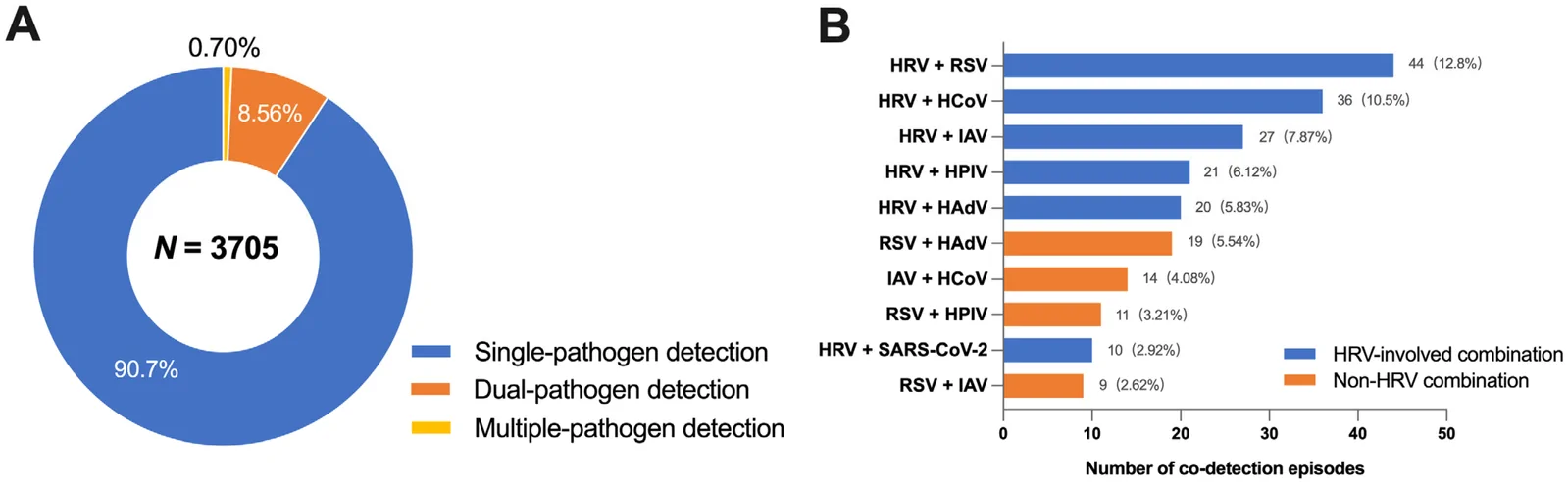
Analysis of co-detection patterns among testing episodes at Wuhan No. 1 Hospital, 2025. (**A**) Donut chart showing the distribution of detection categories among all positive episodes (*n* = 3705). (**B**) Bar chart showing the top 10 most frequent pathogen combinations among co-detection episodes (*n* = 343).

Co-detection of two or more pathogens was identified in 343 positive episodes, accounting for 9.26% of all positive episodes. Dual-pathogen detection accounted for 317 episodes (8.56% of positives), and multiple-pathogen detection (≥3 pathogens) for 26 episodes (0.70%). A total of 59 distinct pathogen combinations were observed. The 10 most frequent combinations comprised 211 episodes (61.5% of all co-detections). HRV plus RSV was the most common pairing (44 episodes, 12.8%), followed by HCoV plus HRV (36 episodes, 10.5%), HRV plus IAV (27 episodes, 7.87%), HPIV plus HRV (21 episodes, 6.12%), HAdV plus HRV (20 episodes, 5.83%), HAdV plus RSV (19 episodes, 5.54%), HCoV plus IAV (14 episodes, 4.08%), HPIV plus RSV (11 episodes, 3.21%), HRV plus SARS-CoV-2 (10 episodes, 2.92%), and IAV plus RSV (9 episodes, 2.62%). HRV participated in 6 of the top 10 combinations (158 episodes), highlighting its central role in co-detection (Figure 6).

### 3.6. Factors Associated with Respiratory Pathogen Detection

Multivariable logistic regression included 12,220 testing episodes (3705 positive and 8515 negative). Two models were fitted: Model 1, including age group, sex, and season (AUC = 0.651), and Model 2, which additionally included clinical department and showed acceptable discrimination (AUC = 0.673) and good calibration (Hosmer–Lemeshow *p* = 0.61). Multicollinearity diagnostics for Model 2 gave dimensionality-adjusted GVIFs of 2.11 for age group (GVIF = 19.9, *Df* = 2) and 1.29 for clinical department (GVIF = 20.5, *Df* = 6), both below the conventional threshold of 5.0, indicating that the standard errors were not materially inflated. The crude association for male sex was weak (OR 1.08, 95% CI 1.00–1.17, *p* = 0.047) and was attenuated after adjustment in both models (Model 1 aOR 1.02, 95% CI 0.94–1.11, *p* = 0.64; Model 2 aOR 0.99, 95% CI 0.91–1.08, *p* = 0.83). A far more pronounced attenuation, however, involved age: compared with adults, children had a strongly elevated crude OR (5.03, 95% CI 4.45–5.68, *p* < 0.0001) that was essentially unchanged in Model 1 (aOR 4.99, 95% CI 4.42–5.65, *p* < 0.0001) but became non-significant in Model 2 after adjustment for clinical department (aOR 1.30, 95% CI 0.82–2.00, *p* = 0.25). This pattern reflects the strong overlap between age group and clinical department: all 1955 pediatric attendees were children, and 94.9% of children attended pediatrics, so that within Model 2 the pediatric contrast is carried almost entirely by the department term. Because the two variables can be contrasted only through the 104 episodes contributed by children tested outside pediatrics, this adjusted estimate is imprecise rather than null: its confidence interval (0.82–2.00) does not exclude a clinically relevant residual effect of age (Supplementary Table S7, Panels C and D).

A post hoc sensitivity analysis directly addressed this issue. Among the 104 children who attended non-pediatric departments, the positivity rate was only 26.9% (28/104, 95% CI 19.3–36.2%), which did not differ significantly from that of adults (23.3%; *χ*^2^ = 0.74, *p* = 0.39) but was far below the 62.2% observed among children attending pediatrics (*χ*^2^ = 51.4, *p* < 0.0001). This finding corroborates the interpretation of Model 2, but its own interpretation is constrained by the strongly skewed age composition of this stratum: 88 of the 104 episodes (84.6%) involved adolescents aged 12–17 years, who had a lower detection rate than younger children even within pediatrics, and only 7 involved children under 6 years (Supplementary Table S7, Panel C). After direct standardization to the age-specific rates of pediatric episodes, the expected positivity in this stratum would have been 47.4% (49.3 of 104 episodes), giving a standardized detection ratio of 0.57 (95% CI 0.38–0.82) (Supplementary Table S7, Panel D). Its lower crude positivity therefore reflects both its age composition and the testing context, and cannot be attributed to either alone. This comparison should be regarded as exploratory: children attending non-pediatric departments may differ from pediatric attendees in underlying conditions, reason for consultation, and clinical severity, and it cannot demonstrate the absence of an age effect.

**Figure 7:**
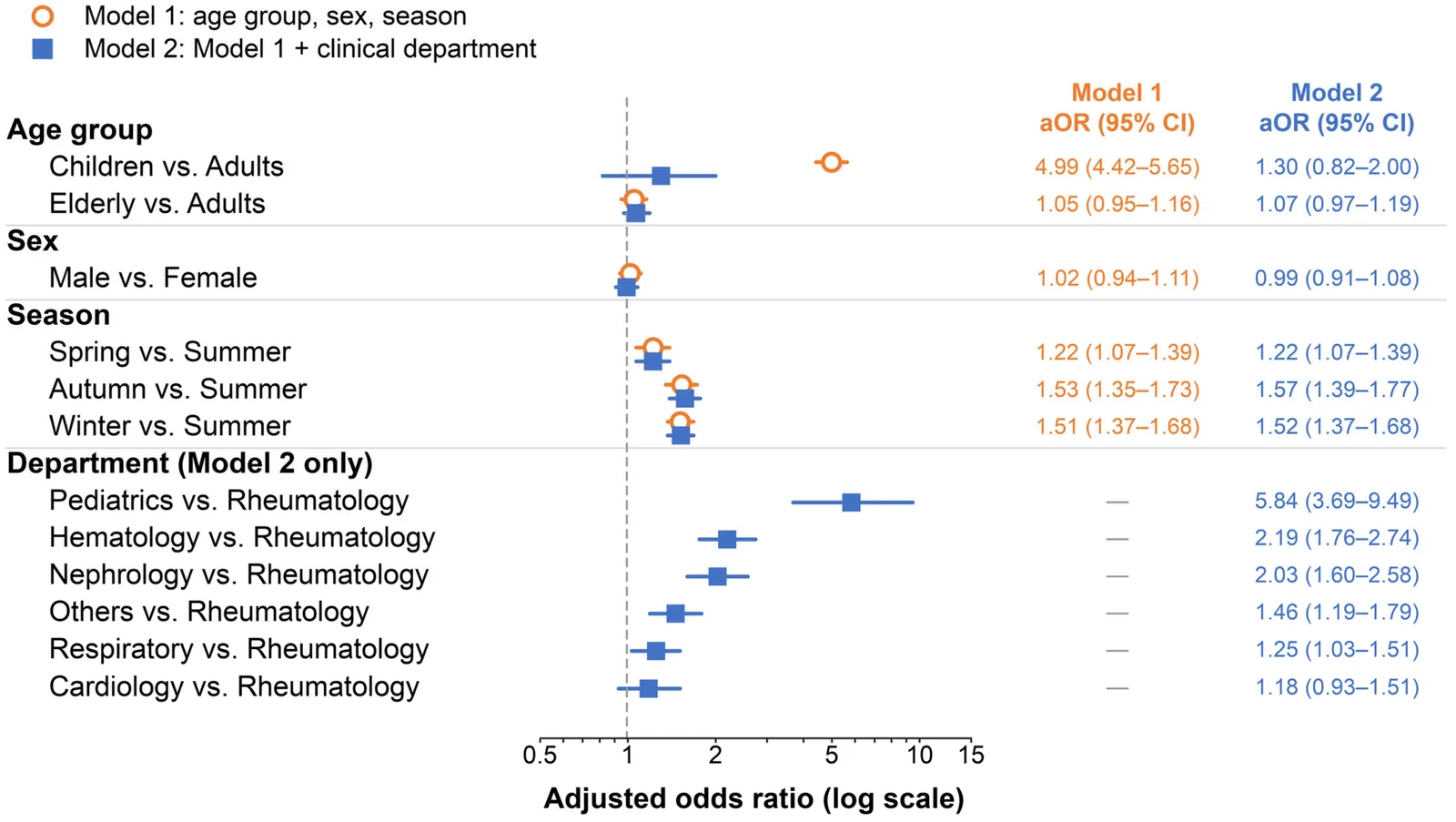
Forest plot of adjusted odds ratios from Models 1 and 2 of multivariate logistic regression analysis of factors associated with respiratory pathogen detection. Open circles represent aORs from Model 1 (age group, sex, and season); filled squares represent aORs from Model 2 (Model 1 plus clinical department); horizontal lines represent 95% confidence intervals (CI). The vertical dashed line indicates OR = 1.0 (no association). Variables with 95% CI not crossing 1.0 were considered statistically significant independent factors. Department variables were included in Model 2 only. Reference categories: Adults (age group), Female (sex), Summer (season), Rheumatology (department).

Clinical department and season were independently associated with pathogen detection. Rheumatology was chosen as the reference category because it had the lowest crude detection rate among the seven department categories (18.7%, 158/845), providing a conservative baseline for the adjusted odds ratios of the other departments. Compared with Rheumatology, pediatric attendance showed the strongest association with pathogen detection (aOR 5.84, 95% CI 3.69–9.49, *p* < 0.0001), followed by Hematology (aOR 2.19, 95% CI 1.76–2.74, *p* < 0.0001), Nephrology (aOR 2.03, 95% CI 1.60–2.58, *p* < 0.0001), Others (aOR 1.46, 95% CI 1.19–1.79, *p* = 0.0003), and Respiratory (aOR 1.25, 95% CI 1.03–1.51, *p* = 0.025). Cardiology was not significantly different from Rheumatology (aOR 1.18, 95% CI 0.93–1.51, *p* = 0.18). Compared with summer, all other seasons were associated with higher odds of pathogen detection: autumn (aOR 1.57, 95% CI 1.39–1.77, *p* < 0.0001), winter (aOR 1.52, 95% CI 1.37–1.68, *p* < 0.0001), and spring (aOR 1.22, 95% CI 1.07–1.39, *p* = 0.0026) (Table 5; Figure 7).

## 4. Discussion

This large retrospective study describes pathogen detection among 12,220 testing episodes, 99.7% of which were ordered during hospitalization at a single tertiary center; the overall detection rate of 30.3% should be interpreted in this context of clinician-driven testing of predominantly hospitalized patients. The overall positivity rate of 30.3% is consistent with recent multicenter surveillance data from China and other regions, which have reported detection rates of 31–62% in patients with acute respiratory symptoms using multiplex PCR [17,18,19]. HRV was the dominant pathogen, accounting for 8.84% of all tested episodes and 29.1% of all positive episodes. This finding aligns with global reports that HRV is the most common viral agent in both community- and hospital-based respiratory infection surveillance [20,21]. A recent study in Guangzhou, China, similarly identified HRV as one of the two leading pathogens among influenza-like illness and severe acute respiratory infection cases during 2024–2025 [17]. The year-round presence of HRV, combined with its role in the majority of common cold episodes, underscores its persistent public health significance. PCR detection of HRV may additionally reflect prolonged post-infectious shedding rather than acute infection, as rhinovirus RNA can be detected for weeks after symptom resolution [20,22].

IAV and RSV ranked second and third among detected pathogens, respectively, reflecting their established roles as major causes of seasonal respiratory disease. The winter IAV positivity rate of 14.0% in our cohort is consistent with the well-documented winter peak of influenza in temperate regions [23]. RSV showed the expected strong age gradient, with a positivity rate of 18.0% in children compared with less than 2.5% in adults and elderly patients. This pattern is in line with international consensus that RSV is a leading cause of acute lower respiratory tract infection in young children, with the highest burden in those under 5 years of age [24,25,26].

The seasonal patterns observed in this study reveal a clear pathogen succession across the year. HRV predominated in spring and autumn, SARS-CoV-2 in summer, and IAV in winter. Autumn HRV activity reached 16.5%, the highest seasonal value for any single pathogen, which may suggest a substantial autumn rhinovirus wave in Wuhan during 2025. However, this observation reflects a single-year snapshot and requires multi-year confirmation. The summer predominance of SARS-CoV-2, contrasting with its marked decline in autumn and winter, is noteworthy. According to national surveillance data from the Chinese Center for Disease Control and Prevention, the predominant SARS-CoV-2 variant circulating in China during the summer of 2025 was NB.1.8.1 (a sixth-generation sub-branch of the XDV variant within the JN.1 lineage), which accounted for approximately 95% of sequenced cases from June through August [27,28,29]. This variant’s immune-escape properties and waning population immunity from the previous winter wave may have driven the summer resurgence [30,31]. Furthermore, the inverse seasonal pattern between SARS-CoV-2 (summer-dominant) and HRV/IAV (autumn/winter-dominant) raises the possibility of viral interference, whereby rhinovirus infection may transiently block SARS-CoV-2 replication through interferon-mediated mechanisms [32], though this hypothesis requires further investigation with longitudinal data. These seasonal shifts have practical implications: clinicians should consider HRV and IAV as leading etiologies during autumn and winter ARI epidemics, respectively, while maintaining awareness of SARS-CoV-2 during summer months.

Age-stratified analysis confirmed that children carried the highest pathogen burden, with an overall positivity rate of 60.4%. This finding is biologically plausible given the immature immune system, limited prior exposure, and close contact in school and daycare settings among young children [3,19]. The “double peak” of HRV and RSV in children is particularly notable and consistent with pediatric surveillance data from eastern and southwestern China [15,19]. Conversely, SARS-CoV-2 positivity was higher in adults and elderly patients than in children, a pattern that has been repeatedly observed in post-pandemic cohorts and may relate to differences in social contact patterns, prior immunity, and receptor expression [31,33].

Co-detection was present in 9.26% of positive episodes (343/3705), a proportion lower than some pediatric and intensive-care studies but consistent with outpatient cohorts [17,34]. HRV was involved in 6 of the 10 most common co-detection pairs, supporting the hypothesis that rhinovirus may act as a “hub pathogen” in respiratory co-detection networks. This pattern could reflect the high baseline prevalence of HRV, but it may also indicate immune-modulatory effects of rhinovirus infection that facilitate concurrent detection of other respiratory viruses [32]. Whether these co-detections represent true simultaneous infections or sequential detection of residual nucleic acids cannot be definitively determined in a cross-sectional design [35]. Molecular co-detection does not establish biological interaction, simultaneous active infection, or the contribution of each detected pathogen to the patient’s symptoms; in particular, HRV and HCoV can be detected for weeks after the acute infectious period, and some co-detections may therefore represent sequential rather than simultaneous infection.

A notable analytical finding of this study is the attenuation of the age effect after multivariable adjustment. In univariate analysis, children had a more than five-fold higher odds of pathogen detection compared with adults, but this association became non-significant after adjusting for clinical department. This finding reflects the near-complete overlap between young age and pediatric attendance in this testing-defined cohort rather than evidence that age is immaterial: because 94.9% of children were tested in pediatrics, the children-versus-adults contrast rests almost entirely on the 104 episodes described above, and its adjusted confidence interval (0.82–2.00) is too wide to exclude a substantial residual effect of age. Within childhood itself, detection decreased markedly with increasing age among pediatric attendees, in whom the testing setting was constant, indicating that age remains an informative determinant even though it cannot be separated from clinical department in the multivariable model. Consistently, the dimensionality-adjusted GVIFs reported in Section 3.6 were below 5.0 for both variables, indicating that this overlap inflated the standard errors only marginally. The attenuation of the age estimate is therefore not a numerical artifact of collinearity but genuine confounding that the available data cannot resolve. From a practical standpoint, clinical department and season are more informative than the crude children-versus-adults categorization for predicting detection in a testing-defined cohort.

The strong independent association of pediatric department attendance (aOR 5.84) likely reflects the concentration of symptomatic children with acute infections in pediatric services, whereas rheumatology and cardiology departments primarily manage chronic or non-infectious conditions. Hematology and nephrology also showed elevated adjusted odds, possibly reflecting immunosuppression, chronic disease, or closer infectious-disease monitoring in these patient groups.

Our findings carry several implications for diagnostic practice and infection control. First, the dominance of HRV and the clear seasonality of IAV and SARS-CoV-2 support the value of multiplex PCR testing in the diagnostic workup of clinically suspected ARI, particularly during high-circulation seasons. Second, the identification of pediatric department attendance as the strongest independent factor associated with pathogen detection emphasizes the need for targeted surveillance and infection-control measures in pediatric healthcare settings. Third, the high pediatric burden of HRV and RSV underscores the potential public health value of future RSV immunization programs and ongoing research into HRV vaccines or antiviral therapies.

This study has several limitations. First, it was a single-center retrospective study, and the findings describe predominantly hospitalized, tested patients and may not generalize to outpatient or primary-care settings, where test-ordering patterns differ substantially. Second, clinical variables were limited to those in the electronic medical record. Third, the multiplex PCR panel included only 12 predefined pathogens and did not cover typical bacterial pathogens, potentially underestimating pathogen diversity. Fourth, viral genotyping or subtyping was not performed. Fifth, seasons were defined by local climatic boundaries; monthly rates are provided to verify robustness. Sixth, comorbidity categories were derived by keyword matching of the free-text diagnosis field, which mixes active problems with past medical history, and should be regarded as approximations; comorbidities were described but not entered into the regression model. Seventh, this study covered only a single calendar year (2025), and the seasonal patterns observed may represent year-to-year variation rather than stable long-term seasonal trends. Eighth, multiplex PCR detects pathogen nucleic acid but cannot distinguish active infection from residual shedding; individual Ct values were not retained in the exported dataset. Ninth, testing was ordered at clinician discretion, and the hospital information system does not retain a structured ordering indication, so the indication for testing could not be verified for individual episodes; reported rates are detection proportions among tested patients. Tenth, the 114 excluded self-paid tests had a higher detection rate and more children, which may have led to underestimation of detection levels, particularly among children.

## 5. Conclusions

Among tested, predominantly hospitalized patients with clinically suspected acute respiratory infection at a tertiary hospital in Wuhan in 2025, HRV was the most frequently detected pathogen, followed by IAV, RSV, and SARS-CoV-2, and detection showed marked seasonal and age-related variation. Detection was independently associated with clinical department and season after adjustment, whereas the apparent association with age did not persist; because young age and pediatric attendance overlapped almost completely in this cohort, the two effects cannot be separated, and a pronounced within-childhood age gradient was nevertheless present. These findings describe pathogen detection among tested inpatients rather than population-level infection rates and should not be extrapolated to outpatient or community settings; they may nonetheless inform local diagnostic stewardship and infection-control practice.

## Figures and Tables

**Table 1:** Demographic characteristics and pathogen detection category distribution of testing episodes with respiratory pathogen multiplex PCR testing at Wuhan No. 1 Hospital, 2025 (*N* = 12,220).

Variable	Category	*n* (%) ^1^
Total testing episodes	-	12,220 (100)
Sex	Male	6589 (53.9)
	Female	5631 (46.1)
Age group	Children	2059 (16.8)
	Adults	3078 (25.2)
	Elderly	7083 (58.0)
Season	Spring	1981 (16.2)
	Summer	3706 (30.3)
	Autumn	2170 (17.8)
	Winter	4363 (35.7)
Detection category	Overall positive	3705 (30.3)
	Single-pathogen detection	3362 (27.5)
	Dual-pathogen detection	317 (2.59)
	Multiple-pathogen detection (≥3)	26 (0.21)
	Negative	8515 (69.7)
Clinical department ^2^	Pediatrics	1955 (16.0)
	Respiratory	4735 (38.7)
	Hematology	1001 (8.19)
	Cardiology	857 (7.01)
	Nephrology	666 (5.45)
	Rheumatology	845 (6.91)
	Others	2161 (17.7)

^1^ Data are presented as *n* (%). Percentages were calculated based on the total number of testing episodes (*N* = 12,220). ^2^ Clinical department distribution and detailed positivity rates by department are presented in Table A1.

**Table 2:** Detection rates of respiratory pathogens among testing episodes for clinically suspected acute respiratory infection at Wuhan No. 1 Hospital, 2025 (*N* = 12,220).

Pathogen ^1^	Abbrev.	Type	Positive (*n*)	Positivity Rate (%) ^2^	95% CI (%) ^3^
Human rhinovirus	HRV	Virus	1080	8.84	8.35–9.35
Influenza A virus	IAV	Virus	646	5.29	4.90–5.70
Respiratory syncytial virus	RSV	Virus	581	4.75	4.39–5.15
SARS-CoV-2	SARS-CoV-2	Virus	505	4.13	3.79–4.50
Human coronavirus	HCoV	Virus	461	3.77	3.45–4.13
Human parainfluenza virus	HPIV	Virus	291	2.38	2.13–2.67
Human adenovirus	HAdV	Virus	209	1.71	1.50–1.96
Human metapneumovirus	hMPV	Virus	188	1.54	1.33–1.77
*Chlamydia pneumoniae*	CP	Bacteria	52	0.43	0.32–0.56
Human bocavirus	HBoV	Virus	46	0.38	0.28–0.50
*Mycoplasma pneumoniae*	MP	Bacteria	12	0.10	0.06–0.17
Influenza B virus	IBV	Virus	3	0.02	0.01–0.07
Total positive (any pathogen)	-	-	3705 ^4^	30.3	29.5–31.1

^1^ Pathogens are listed in descending order of positivity rate. ^2^ Positivity rate was calculated as the number of positive cases for each pathogen divided by the total number of testing episodes (*N* = 12,220). ^3^ 95% confidence intervals for positivity rates were calculated using the Wilson score method. ^4^ The sum of individual pathogen positive cases exceeds the total positive cases (*n* = 3705) due to co-detections.

**Table 3:** Seasonal distribution of respiratory pathogen detection and dominant pathogens among testing episodes at Wuhan No. 1 Hospital, 2025.

Season ^1^	Total Cases (*n*)	Positive Cases (*n*)	Positivity Rate (%)	Dominant Pathogen ^2^	Positivity Rate of Dominant Pathogen (%)
Spring	1981	579	29.2	HRV	9.59
Summer	3706	941	25.4	SARS-CoV-2	7.93
Autumn	2170	741	34.1	HRV	16.5
Winter	4363	1444	33.1	IAV	14.0
Total	12,220	3705	30.3	-	-

^1^ Seasons were defined as described in the Methods section. ^2^ The dominant pathogen was defined as the pathogen with the highest positivity rate within each season.

**Table 4:** Age-stratified distribution of respiratory pathogen detection and dominant pathogens among testing episodes at Wuhan No. 1 Hospital, 2025.

Age Group ^1^	Total Cases (*n*)	Positive Cases (*n*)	Positivity Rate (%)	Dominant Pathogen ^2^	Dominant Pathogen Rate (%)
Children	2059	1244	60.4	HRV	19.8
Adults	3078	717	23.3	HRV	6.01
Elderly	7083	1744	24.6	HRV	6.88
Total	12,220	3705	30.3	-	-

^1^ Age groups were defined as: Children (<18 years), Adults (18–59 years), and Elderly (≥60 years). ^2^ The dominant pathogen was defined as the pathogen with the highest positivity rate within each age group.

**Table 5:** Univariate and multivariate logistic regression analysis of factors associated with respiratory pathogen detection among testing episodes at Wuhan No. 1 Hospital, 2025.

Variable	Category	Crude OR (95% CI) ^1^	*p* Value	Model 1 aOR (95% CI) ^2,3^	*p* Value	Model 2 aOR (95% CI) ^2,3^	*p* Value ^4^
Age group	Children vs. Adults	5.03 (4.45–5.68)	<0.0001 ****	4.99 (4.42–5.65)	<0.0001 ****	1.30 (0.82–2.00)	0.25
	Elderly vs. Adults	1.08 (0.97–1.19)	0.15	1.05 (0.95–1.16)	0.32	1.07 (0.97–1.19)	0.20
	Adults (ref.)	1.00 (reference)	-	1.00 (reference)	-	1.00 (reference)	-
Sex	Male vs. Female	1.08 (1.00–1.17)	0.047 *	1.02 (0.94–1.11)	0.64	0.99 (0.91–1.08)	0.83
	Female (ref.)	1.00 (reference)	-	1.00 (reference)	-	1.00 (reference)	-
Season	Spring vs. Summer	1.21 (1.07–1.37)	0.0019 **	1.22 (1.07–1.39)	0.0022 **	1.22 (1.07–1.39)	0.0026 **
	Autumn vs. Summer	1.52 (1.36–1.71)	<0.0001 ****	1.53 (1.35–1.73)	<0.0001 ****	1.57 (1.39–1.77)	<0.0001 ****
	Winter vs. Summer	1.45 (1.32–1.60)	<0.0001 ****	1.51 (1.37–1.68)	<0.0001 ****	1.52 (1.37–1.68)	<0.0001 ****
	Summer (ref.)	1.00 (reference)	-	1.00 (reference)	-	1.00 (reference)	-
Department	Pediatrics vs. Rheumatology	7.16 (5.90–8.72)	<0.0001 ****	-	-	5.84 (3.69–9.49)	<0.0001 ****
	Hematology vs. Rheumatology	2.15 (1.73–2.67)	<0.0001 ****	-	-	2.19 (1.76–2.74)	<0.0001 ****
	Nephrology vs. Rheumatology	2.02 (1.59–2.56)	<0.0001 ****	-	-	2.03 (1.60–2.58)	<0.0001 ****
	Others vs. Rheumatology	1.46 (1.20–1.78)	0.0002 ***	-	-	1.46 (1.19–1.79)	0.0003 ***
	Respiratory vs. Rheumatology	1.25 (1.04–1.50)	0.020 *	-	-	1.25 (1.03–1.51)	0.025 *
	Cardiology vs. Rheumatology	1.25 (0.99–1.58)	0.067	-	-	1.18 (0.93–1.51)	0.18
	Rheumatology (ref.)	1.00 (reference)	-	-	-	1.00 (reference)	-

^1^ Crude ORs were calculated using univariate logistic regression models, with each independent variable entered separately. ^2^ Adjusted ORs were calculated using two multivariable logistic regression models: Model 1 included age group, sex, and season; Model 2 additionally included clinical department. The area under the ROC curve was 0.651 for Model 1 and 0.673 for Model 2; Model 2 showed good calibration (Hosmer–Lemeshow test *p* = 0.61). ^3^ Reference categories: Age group = Adults; Sex = Female; Season = Summer; Clinical department = Rheumatology. ^4^ *p* value markers: * *p* < 0.05; ** *p* < 0.01; *** *p* < 0.001; **** *p* < 0.0001. Total observations: *N* = 12,220 testing episodes (Positive = 3705; Negative = 8515).

## Data Availability

The data presented in this study are available on request from the corresponding author (The data are not publicly available due to privacy and ethical restrictions).

## Supplementary Materials

The following supporting information can be downloaded at: https://www.mdpi.com/article/10.3390/pathogens15101064/s1, Table S1: Testing episodes and pathogen detection by encounter type and specimen type; Table S2: Recorded diagnoses grouped into four mutually exclusive categories and pathogen detection within each category, Wuhan, China, 2025; Table S3: Comorbidity profile of the cohort by age group, derived from recorded diagnoses, Wuhan, China, 2025; Table S4: Sensitivity analysis restricted to each patient’s first testing episode: comparison with the episode-level analysis, Wuhan, China, 2025; Table S5: Detection of 12 respiratory pathogens by season among 12,220 testing episodes for clinically suspected acute respiratory infection, Wuhan, China, 2025; Table S6: Detection of 12 respiratory pathogens by age group among 12,220 testing episodes, Wuhan, China, 2025; Table S7: Detection among children (<18 years) by age band and testing department, with age-standardized comparison between pediatric and non-pediatric settings, Wuhan, China, 2025.

## Abbreviations

The following abbreviations are used in this manuscript:

aOR	adjusted odds ratio
CI	confidence interval
CP	*Chlamydia pneumoniae*
HAdV	human adenovirus
HBoV	human bocavirus
HCoV	human coronavirus
hMPV	human metapneumovirus
HPIV	human parainfluenza virus
HRV	human rhinovirus
IAV	influenza A virus
IBV	influenza B virus
MP	*Mycoplasma pneumoniae*
OR	odds ratio
RSV	respiratory syncytial virus
SARS-CoV-2	severe acute respiratory syndrome coronavirus 2
ARI	acute respiratory infection

## Appendix A

**Table A1:** Distribution of respiratory pathogen positivity by clinical department testing episodes at Wuhan No. 1 Hospital, 2025.

Department ^1^	Total (*n*)	Positive (*n*)	Positivity Rate (%) ^2^
Pediatrics	1955	1216	62.2
Hematology	001	331	33.1
Nephrology	666	211	31.7
Cardiology	857	191	22.3
Respiratory	4735	1055	22.3
Rheumatology	845	158	18.7
Others	2161	543	25.1
Total	12,220	3705	30.3

^1^ Departments are listed in descending order of detection rate, with Others retained at the end. ^2^ Positivity rate (%) = number of positive cases/total cases in each department × 100.

**Table A2:** Seasonal distribution of 12 respiratory pathogens among testing episodes at Wuhan No. 1 Hospital, 2025.

Pathogen ^1^	Spring *n* (%) ^2,3^	Summer *n* (%)	Autumn *n* (%)	Winter *n* (%)
HRV	190 (9.59)	182 (4.91)	357 (16.5)	351 (8.04)
IAV	0 (0.00)	2 (0.05)	35 (1.61)	609 (14.0)
RSV	93 (4.69)	126 (3.40)	203 (9.35)	159 (3.64)
SARS-CoV-2	156 (7.87)	294 (7.93)	26 (1.20)	29 (0.66)
HCoV	14 (0.71)	199 (5.37)	117 (5.39)	131 (3.00)
HPIV	43 (2.17)	111 (3.00)	56 (2.58)	81 (1.86)
HAdV	45 (2.27)	58 (1.57)	30 (1.38)	76 (1.74)
hMPV	60 (3.03)	11 (0.30)	13 (0.60)	104 (2.38)
CP	17 (0.86)	10 (0.27)	4 (0.18)	21 (0.48)
HBoV	0 (0.00)	22 (0.59)	11 (0.51)	13 (0.30)
MP	3 (0.15)	6 (0.16)	0 (0.00)	3 (0.07)
IBV	0 (0.00)	0 (0.00)	2 (0.09)	1 (0.02)
Total ^4^	579 (29.2)	941 (25.4)	741 (34.1)	1444 (33.1)

^1^ Pathogens are listed in descending order of overall detection rate. ^2^ Percentages in pathogen rows were calculated using the total number of testing episodes within each season as the denominator. ^3^ Seasons were defined as described in Section 2.4. ^4^ The Total row represents the number of episodes positive for at least one pathogen; sums of individual pathogens exceed these totals because of co-detections.
