# Supplementary material for: Respiratory Pathogen Spectrum Among 12,220 Testing Episodes for Clinically Suspected Acute Respiratory Infection in Predominantly Hospitalized Patients at a Tertiary Hospital in Wuhan, China, 2025: A Retrospective Cross-Sectional Study
Source: Pathogens. 2026 Oct 8;15(10):1064. doi: 10.3390/pathogens15101064 (PMC13708373; doi:10.3390/pathogens15101064)
Supplement: Supplementary file 1 [file pathogens-15-01064-s001.zip › pathogens-4565361-supplementary.pdf]

Supplementary Materials

(Tables S1 – S7)

Table S1. Testing episodes and pathogen detection by encounter type and specimen type, Wuhan, China, 2025

Characteristic	*n*	% of episodes	No. with detection	Detection %
Encounter type				
Inpatient	12,189	99.7	3696	30.3
Outpatient	28	0.23	8	28.6
Emergency	3	0.02	1	33.3
Specimen type				
Oropharyngeal swab	12,196	99.8	3697	30.3
Nasal swab	24	0.20	8	33.3
Total	12,220	100.0	3705	30.3

Notes: Encounter type (inpatient admission, outpatient clinic, or emergency department) was retrieved from the hospital information system at the time of test ordering. Detection proportions were similar across encounter types (30.3%, 28.6%, and 33.3% for inpatient, outpatient, and emergency episodes, respectively); the very small numbers of outpatient (*n* = 28) and emergency-department (*n* = 3) episodes preclude formal between-group comparison. The 24 nasal swabs were collected in the cardiac intensive care unit (*n* = 10), the two hematology wards (*n* = 8), and six other adult wards (*n* = 6); all nasal-swab episodes involved adult or elderly patients. Specimen type therefore did not vary systematically by age group.

Table S2. Recorded diagnoses grouped into four mutually exclusive categories and pathogen detection within each category, Wuhan, China, 2025

Diagnostic category	*n*	% of episodes	No. with detection	Detection %	Children <18 y, % of category
Lower respiratory tract infection (LRTI)-related diagnosis	7531	61.6	2529	33.6	17.6
Upper respiratory tract infection (URTI)-related diagnosis without LRTI codes	839	6.9	330	39.3	57.4
Other respiratory presentations	1394	11.4	335	24.0	5.5
No respiratory term recorded in the diagnosis field	2456	20.1	511	20.8	7.1
Total	12,220	100.0	3705	30.3	16.9

Notes: Each testing episode was assigned to the first matching category in a fixed hierarchy (LRTI-related > URTI-related > other respiratory presentations > no respiratory term), so that the four categories are mutually exclusive and sum to 12,220.

Category definitions are based on Chinese diagnosis terms in the free-text diagnosis field: LRTI-related: pneumonia, bronchitis, pulmonary infection, lower respiratory tract infection, respiratory failure, pleural effusion, bronchiectasis, emphysema; URTI-related: upper respiratory tract infection, pharyngitis, tonsillitis, common cold, rhinitis, sinusitis, laryngitis, epiglottitis; other respiratory presentations: unspecified respiratory infection, fever, cough, expectoration, dyspnea, wheeze, chest tightness, asthma, chronic obstructive pulmonary disease, or any other diagnosis containing a Chinese term for a respiratory symptom or structure. The categories are approximations derived from free text, which mixes active problems with past medical history; they are reported to characterize the cohort (a testing-defined cohort in which testing was ordered for clinically suspected acute respiratory infection) and were not used as covariates in the regression models. Detection was highest in episodes carrying an URTI code without LRTI codes (39.3%), which were predominantly children (57.4% of that category); this reflects age-related testing patterns and should be interpreted with caution.

The fourth category is labeled 'no respiratory term recorded' because the diagnosis field is a free-text field that captures mainly the principal diagnosis—frequently a traditional Chinese medicine syndrome pattern or a chronic non-respiratory condition—whereas presenting symptoms such as fever and cough are recorded in the presenting complaint and progress notes. Episodes in this category were almost exclusively inpatient admissions, were concentrated in non-respiratory departments (rheumatology, hematology, cardiology, gastroenterology, nephrology), and were distributed evenly across the calendar year; their detection rate of 20.8% indicates that most were tested on a clinical suspicion of respiratory infection that was simply not captured in the diagnosis field. The category should therefore be read as reflecting incomplete diagnostic recording rather than an absence of clinical suspicion.

Table S3. Comorbidity profile of the cohort by age group, derived from recorded diagnoses, Wuhan, China, 2025

Comorbidity category	Children <18 y (*n* = 2059)	Adults 18–59 y (*n* = 3078)	Elderly ≥60 y (*n* = 7083)	All episodes (*n* = 12,220)
Heart failure or coronary artery disease	—	335 (10.9%) / 29.9%	2274 (32.1%) / 26.2%	2609 (21.4%) / 26.6%
Chronic obstructive pulmonary disease or asthma	20 (1.0%) / 65.0%	221 (7.2%) / 30.3%	1986 (28.0%) / 25.6%	2227 (18.2%) / 26.4%
Diabetes mellitus	2 (0.1%) / 0.0%	275 (8.9%) / 28.0%	1587 (22.4%) / 25.7%	1864 (15.3%) / 26.0%
Chronic kidney disease or renal failure	1 (0.0%) / 0.0%	337 (10.9%) / 26.1%	1286 (18.2%) / 28.7%	1624 (13.3%) / 28.1%
Immunosuppression or autoimmune disease	13 (0.6%) / 23.1%	492 (16.0%) / 27.4%	429 (6.1%) / 24.9%	934 (7.6%) / 26.2%
Chronic liver disease	10 (0.5%) / 30.0%	285 (9.3%) / 28.1%	603 (8.5%) / 24.5%	898 (7.3%) / 25.7%
Solid tumour	—	157 (5.1%) / 20.4%	721 (10.2%) / 19.6%	878 (7.2%) / 19.7%
Hematologic malignancy	5 (0.2%) / 40.0%	316 (10.3%) / 41.8%	472 (6.7%) / 32.2%	793 (6.5%) / 36.1%
≥1 comorbidity category	49 (2.4%) / 40.8%	1610 (52.3%) / 27.0%	5328 (75.2%) / 25.2%	6987 (57.2%) / 25.8%
No comorbidity category recorded	2010 (97.6%) / 60.9%	1468 (47.7%) / 19.2%	1755 (24.8%) / 22.7%	5233 (42.8%) / 36.4%

Notes: Data are *n* (% of the age group) / detection %. Detection % is the proportion of episodes in the cell with detection of at least one of the 12 panel targets. Em dashes indicate no episode in the cell. Comorbidity categories were derived by keyword matching of the free-text diagnosis field in the electronic medical record, which mixes active problems with past medical history; they should therefore be regarded as approximations rather than validated comorbidity definitions. Categories are not mutually exclusive; patients may appear in more than one category, whereas the last two rows are mutually exclusive and exhaustive. In unstratified data, detection appeared lower among episodes with at least one comorbidity category (25.8%) than among those with none (36.4%); this reflects Simpson's paradox arising from age confounding, because children—who had the highest detection rate (60.4%) and almost no recorded comorbidities—dominate the 'no comorbidity' stratum. Within the adult and elderly strata, detection was higher among episodes with a recorded comorbidity (27.0% vs. 19.2% and 25.2% vs. 22.7%, respectively).

Table S4. Sensitivity analysis restricted to each patient's first testing episode: comparison with the episode-level analysis, Wuhan, China, 2025.

**Analysis stratum**	**All testing episodes (*n* = 12,220) no. positive / N**	**Detection % (95% CI)**	**First episode per patient (*n* = 10,028) no. positive / *N***	**Detection % (95% CI)**	**Difference (percentage points)**
**Panel A. Detection of at least one target**					
Overall	3705 / 12,220	30.3 (29.5–31.1)	3048 / 10,028	30.4 (29.5–31.3)	+0.08
Season: Spring	579 / 1981	29.2 (27.3–31.3)	484 / 1663	29.1 (27.0–31.3)	−0.12
Season: Summer	941 / 3706	25.4 (24.0–26.8)	749 / 2965	25.3 (23.7–26.9)	−0.13
Season: Autumn	741 / 2170	34.1 (32.2–36.2)	584 / 1679	34.8 (32.5–37.1)	+0.64
Season: Winter	1444 / 4363	33.1 (31.7–34.5)	1231 / 3721	33.1 (31.6–34.6)	−0.01
Children (<18 y)	1244 / 2059	60.4 (58.3–62.5)	1148 / 1901	60.4 (58.2–62.6)	−0.03
Adults (18–59 y)	717 / 3078	23.3 (21.8–24.8)	557 / 2579	21.6 (20.1–23.2)	−1.70
Elderly (≥60 y)	1744 / 7083	24.6 (23.6–25.6)	1343 / 5548	24.2 (23.1–25.4)	−0.42
Male	2048 / 6589	31.1 (30.0–32.2)	1657 / 5277	31.4 (30.2–32.7)	+0.32
Female	1657 / 5631	29.4 (28.3–30.6)	1391 / 4751	29.3 (28.0–30.6)	−0.15
**Panel B. Pathogen-specific detection (targets are not mutually exclusive)**					
HRV	1080 / 12,220	8.84 (8.35–9.35)	852 / 10,028	8.50 (7.97–9.06)	−0.34
IAV	646 / 12,220	5.29 (4.90–5.70)	590 / 10,028	5.88 (5.44–6.36)	+0.60
RSV	581 / 12,220	4.75 (4.39–5.15)	473 / 10,028	4.72 (4.32–5.15)	−0.04
SARS-CoV-2	505 / 12,220	4.13 (3.79–4.50)	392 / 10,028	3.91 (3.55–4.31)	−0.22
HCoV	461 / 12,220	3.77 (3.45–4.13)	338 / 10,028	3.37 (3.03–3.74)	−0.40
HPIV	291 / 12,220	2.38 (2.13–2.67)	253 / 10,028	2.52 (2.23–2.85)	+0.14
HAdV	209 / 12,220	1.71 (1.50–1.96)	186 / 10,028	1.85 (1.61–2.14)	+0.14
hMPV	188 / 12,220	1.54 (1.33–1.77)	169 / 10,028	1.69 (1.45–1.96)	+0.15
CP	52 / 12,220	0.43 (0.32–0.56)	51 / 10,028	0.51 (0.39–0.67)	+0.08
HBoV	46 / 12,220	0.38 (0.28–0.50)	39 / 10,028	0.39 (0.28–0.53)	+0.01
MP	12 / 12,220	0.10 (0.06–0.17)	11 / 10,028	0.11 (0.06–0.20)	+0.01
IBV	3 / 12,220	0.02 (0.01–0.07)	2 / 10,028	0.02 (0.01–0.07)	0.00
**Panel C. Monthly detection of at least one target**					
January	452 / 1291	35.0 (32.5–37.7)	433 / 1220	35.5 (32.9–38.2)	+0.48
February	224 / 919	24.4 (21.7–27.3)	193 / 812	23.8 (21.0–26.8)	−0.61
March	274 / 987	27.8 (25.1–30.6)	234 / 855	27.4 (24.5–30.5)	−0.39
April	276 / 967	28.5 (25.8–31.5)	235 / 813	28.9 (25.9–32.1)	+0.36
May	304 / 985	30.9 (28.1–33.8)	247 / 810	30.5 (27.4–33.8)	−0.37
June	215 / 931	23.1 (20.5–25.9)	187 / 758	24.7 (21.7–27.9)	+1.58
July	281 / 895	31.4 (28.4–34.5)	211 / 721	29.3 (26.1–32.7)	−2.13
August	176 / 818	21.5 (18.8–24.5)	142 / 649	21.9 (18.9–25.2)	+0.36
September	209 / 905	23.1 (20.5–25.9)	166 / 709	23.4 (20.4–26.7)	+0.32
October	339 / 1047	32.4 (29.6–35.3)	266 / 813	32.7 (29.6–36.0)	+0.34
November	447 / 1138	39.3 (36.5–42.1)	342 / 855	40.0 (36.8–43.3)	+0.72
December	508 / 1337	38.0 (35.4–40.6)	392 / 1013	38.7 (35.7–41.7)	+0.70
Co-detection of ≥2 targets (denominator = positive episodes)	343 / 3705	9.26	287 / 3048	9.42	+0.16

Notes: Patients were uniquely identified by their hospital registration number, which is assigned once and remains unchanged across repeated admissions. "First episode per patient" denotes the earliest testing episode recorded under a given registration number; this analysis retained one episode per patient (*n* = 10,028) and excluded 2192 repeat episodes

contributed by 1300 patients (13.0%). Detection % is the percentage of episodes in the row stratum with detection of at least one of the 12 panel targets (Panels A and C) or of the specified pathogen (Panel B). 95% CIs are Wilson score intervals. Difference = first-episode percentage minus all-episode percentage, expressed in percentage points. Season, age group, and sex rows each partition the corresponding analysis set, whereas pathogen rows do not (one episode may yield more than one target). In the final row the denominator is restricted to positive episodes. Across all panels the two analyses agreed closely: all differences were ≤2.13 percentage points, all 12 pathogens retained their original rank order, and the November maximum (40.0%) and August minimum (21.9%) were preserved.

Table S5. Detection of 12 respiratory pathogens by season among 12,220 testing episodes for clinically suspected acute respiratory infection, Wuhan, China, 2025

Pathogen	Spring (n = 1981)	Summer (n = 3706)	Autumn (n = 2170)	Winter (n = 4363)	χ^2	p	q (BH)
Human rhinovirus (HRV)	190 (9.59%)	182 (4.91%)	357 (16.5%)	351 (8.04%)	232	< 0.001	< 0.001 *
Influenza A virus (IAV)	0 (0.00%)	2 (0.05%)	35 (1.61%)	609 (14.0%)	1,030	< 0.001	< 0.001 *
Respiratory syncytial virus (RSV)	93 (4.69%)	126 (3.40%)	203 (9.35%)	159 (3.64%)	128	< 0.001	< 0.001 *
SARS coronavirus 2 (SARS-CoV-2)	156 (7.87%)	294 (7.93%)	26 (1.20%)	29 (0.66%)	385	< 0.001	< 0.001 *
Human coronavirus (HCoV)	14 (0.71%)	199 (5.37%)	117 (5.39%)	131 (3.00%)	100	< 0.001	< 0.001 *
Human parainfluenza virus (HPIV)	43 (2.17%)	111 (3.00%)	56 (2.58%)	81 (1.86%)	11.9	0.008	0.010 *
Human adenovirus (HAdV)	45 (2.27%)	58 (1.57%)	30 (1.38%)	76 (1.74%)	5.59	0.133	0.160
Human metapneumovirus (hMPV)	60 (3.03%)	11 (0.30%)	13 (0.60%)	104 (2.38%)	100.0	< 0.001	< 0.001 *
Chlamydia pneumoniae (CP)	17 (0.86%)	10 (0.27%)	4 (0.18%)	21 (0.48%)	14.2	0.003	0.005 *
Human bocavirus (HBoV)	0 (0.00%)	22 (0.59%)	11 (0.51%)	13 (0.30%)	13.8	0.003	0.005 *
Mycoplasma pneumoniae (MP)	3 (0.15%)	6 (0.16%)	0 (0.00%)	3 (0.07%)	4.62	0.173	0.182
Influenza B virus (IBV)	0 (0.00%)	0 (0.00%)	2 (0.09%)	1 (0.02%)	5.44	0.182	0.182
Any pathogen (≥1 target)	579 (29.2%)	941 (25.4%)	741 (34.1%)	1,444 (33.1%)	74.7	< 0.001	—

Notes: Data are n (%) of testing episodes positive for the specified pathogen among all episodes tested in the corresponding season (seasonal denominators in column headings). A testing episode was considered positive if any of the 12 panel targets was interpreted as positive according to the manufacturer's instructions (Ct ≤ 40). Seasons were defined according to the local climatic season division: spring, 15 March–15 May; summer, 16 May–19 September; autumn, 20 September–20 November; winter, 21 November–14 March. p values are from Pearson's chi-square tests comparing detection of each pathogen across the four seasons (2 × 4 tables, $df = 3$), except for *M. pneumoniae* and influenza B virus, whose minimum expected cell counts were below 5, for which Fisher's exact test was used instead; q values are p values adjusted across the 12 pathogens using the Benjamini–Hochberg false discovery rate (FDR) procedure. * $q < 0.05$. After FDR correction, nine of the 12 pathogens showed significant seasonal variation. For pathogens with very few detections (e.g., *M. pneumoniae*, influenza B virus), chi-square results should be interpreted with caution; 95% CIs for these pathogens, calculated with the Wilson score method, are reported in Table 2 of the main text.

Table S6. Detection of 12 respiratory pathogens by age group among 12,220 testing episodes, Wuhan, China, 2025

Pathogen	Children (n = 2059)	Adults (n = 3078)	Elderly (n = 7083)	χ^2	p	q (BH)
Human rhinovirus (HRV)	408 (19.8%)	185 (6.01%)	487 (6.88%)	372	< 0.001	< 0.001 *
Influenza A virus (IAV)	176 (8.55%)	113 (3.67%)	357 (5.04%)	60.6	< 0.001	< 0.001 *
Respiratory syncytial virus (RSV)	370 (18.0%)	56 (1.82%)	155 (2.19%)	956	< 0.001	< 0.001 *
SARS coronavirus 2 (SARS-CoV-2)	35 (1.70%)	150 (4.87%)	320 (4.52%)	37.7	< 0.001	< 0.001 *
Human coronavirus (HCoV)	69 (3.35%)	130 (4.22%)	262 (3.70%)	2.84	0.242	0.242
Human parainfluenza virus (HPIV)	160 (7.77%)	36 (1.17%)	95 (1.34%)	310	< 0.001	< 0.001 *
Human adenovirus (HAdV)	118 (5.73%)	31 (1.01%)	60 (0.85%)	238	< 0.001	< 0.001 *
Human metapneumovirus (hMPV)	63 (3.06%)	40 (1.30%)	85 (1.20%)	38.0	< 0.001	< 0.001 *
Chlamydia pneumoniae (CP)	41 (1.99%)	9 (0.29%)	2 (0.03%)	147	< 0.001	< 0.001 *
Human bocavirus (HBoV)	42 (2.04%)	2 (0.06%)	2 (0.03%)	183	< 0.001	< 0.001 *
Mycoplasma pneumoniae (MP)	7 (0.34%)	5 (0.16%)	0 (0.00%)	20.5	< 0.001	< 0.001 *
Influenza B virus (IBV)	0 (0.00%)	2 (0.06%)	1 (0.01%)	2.87	0.234	0.242
Any pathogen (≥1 target)	1244 (60.4%)	717 (23.3%)	1744 (24.6%)	1060	< 0.001	—

Notes: Data are *n* (%) of testing episodes positive for the specified pathogen among all episodes tested in the corresponding age group (denominators in column headings); positivity criteria as in Table S1. Age groups: children (<18 years), adults (18–59 years), and elderly patients (≥60 years). p values are from Pearson's chi-square tests comparing detection of each pathogen across the three age groups (2 × 3 tables, df = 2), except for *M. pneumoniae* and influenza B virus, whose minimum expected cell counts were below 5, for which Fisher's exact test was used instead; q values are Benjamini–Hochberg FDR-adjusted p values across the 12 pathogens. * $q < 0.05$. After FDR correction, ten of the 12 pathogens showed significant age-related variation.

Table S7. Detection among children (<18 years) by age band and testing department, with age-standardized comparison between pediatric and non-pediatric settings, Wuhan, China, 2025

Age band	*n*	No. with detection	Detection %, (95% CI)
**Panel A. All children (*n* = 2059)**			
<1 year	213	142	66.7 (60.1–72.7)
1–2 years	543	381	70.2 (66.2–73.9)
3–5 years	607	403	66.4 (62.5–70.0)
6–11 years	529	263	49.7 (45.5–54.0)
12–17 years	167	55	32.9 (26.3–40.4)
All children	2059	1244	60.4 (58.3–62.5)
**Panel B. Children tested in pediatric departments (*n* = 1955)**			
<1 year	212	142	67.0 (60.4–73.0)
1–2 years	540	378	70.0 (66.0–73.7)
3–5 years	604	401	66.4 (62.5–70.0)
6–11 years	520	259	49.8 (45.5–54.1)
12–17 years	79	36	45.6 (35.0–56.5)
All pediatric episodes	1955	1216	62.2 (60.0–64.3)
**Panel C. Children tested in departments other than pediatrics (*n* = 104)**			
<1 year	1	0	0.00 (0.00–79.3)
1–2 years	3	3	100.0 (43.9–100.0)
3–5 years	3	2	66.7 (20.8–93.9)
6–11 years	9	4	44.4 (18.9–73.3)
12–17 years	88	19	21.6 (14.3–31.3)
All non-pediatric episodes	104	28	26.9 (19.3–36.2)
**Panel D. Direct standardization of Panel C to the age-specific rates of Panel B**			
Observed positives	104	28	26.9 (19.3–36.2)
Expected positives under the Panel B rates	104	49.3	47.4
Standardized detection ratio (95% CI)	—	—	0.57 (0.38–0.82)

Notes: The table is decomposed by testing department: Panel A reports all children, Panel B those tested in pediatric departments and Panel C those tested in other departments, so that Panels B and C sum exactly to Panel A. Within Panel B, in which the testing setting was constant, detection declined across the five ordered bands (Cochran–Armitage χ^2 for trend = 45.7, $df = 1$, $p < 0.0001$). Panel C was strongly skewed toward adolescents: 88 of its 104 episodes (84.6%) involved children aged 12–17 years and only 7 involved children under 6 years, so its crude positivity is strongly influenced by age composition. After direct standardization to the age-specific rates of Panel B, 49.3 positive episodes would have been expected in Panel C whereas 28 were observed, giving a standardized detection ratio of 0.57 (95% CI 0.38–0.82), the interval being derived from the Poisson distribution of the observed count. Because age composition and testing setting vary together in this comparison, the standardized ratio indicates that both contribute to the lower crude positivity in Panel C rather than isolating the effect of either. 95% CIs are Wilson score intervals.
